# Critical role of lipid membranes in polarization and migration of cells: a biophysical view

**DOI:** 10.1007/s12551-021-00781-1

**Published:** 2021-01-11

**Authors:** Erich Sackmann, Motomu Tanaka

**Affiliations:** 1 Physics Department E22/E27, Technical University of Munich, James-Franck-Strasse, 85747 Garching, Germany; 2 Physical Chemistry of Biosystems, Institute of Physical Chemistry, Heidelberg University, 69120 Heidelberg, Germany; 3 Center for Integrative Medicine and Physics, Institute for Advanced Study, Kyoto University, Kyoto, 606-8501 Japan

**Keywords:** Lipid membranes, Cell adhesion, Cell polarization, Cell migration

## Abstract

Cell migration plays vital roles in many biologically relevant processes such as tissue morphogenesis and cancer metastasis, and it has fascinated biophysicists over the past several decades. However, despite an increasing number of studies highlighting the orchestration of proteins involved in different signaling pathways, the functional roles of lipid membranes have been essentially overlooked. Lipid membranes are generally considered to be a functionless two-dimensional matrix of proteins, although many proteins regulating cell migration gain functions only after they are recruited to the membrane surface and self-organize their functional domains. In this review, we summarize how the logistical recruitment and release of proteins to and from lipid membranes coordinates complex spatiotemporal molecular processes. As predicted from the classical framework of the Smoluchowski equation of diffusion, lipid/protein membranes serve as a 2D reaction hub that contributes to the effective and robust regulation of polarization and migration of cells involving several competing pathways.

## Introduction: cell migration driven by membrane protrusion/retraction

Directional migration (crawling) of eukaryotic cells is one of the most relevant processes not only for simple, unicellular organisms like amoeba but also for highly developed metazoans such as mammals (Abercrombie 1980). In general, cell migration can be categorized into two groups: mesenchymal migration and amoeboid movement. Mesenchymal migration is characterized by the formation of actin-containing protrusions, such as lamellipodia, near the spreading front that is followed by retraction of the trailing end. Amoeboid migration is driven by the extension of protrusions at the front side, called pseudopods, which pull cells forward. Pseudopods might consist of actin-free blebs and lamellipodia-like structures, such as uropods of hematopoietic stem cells or invadopodia of cancer cells.

From the viewpoint of nonequilibrium statistical physics, cell migration is also an interesting subject because the front-rear asymmetry is caused by spontaneous symmetry breaking. A simple equation describing instability-driven motion for the movement of a spherical droplet, or a circle in a two-dimensional (2D) projection), including the velocity of center of mass *v*, friction *γ* and the deformation tensor *S*, d*v*_*i*_/d*t* = *γv*_*i*_ − ***v***^2^*v*_*i*_ − *aS*_*ij*_*v*_*j*_, is not sufficient to describe cell migration. Cells adhere to the contact surface, such as the extracellular matrix or other cells, generate forces and actively deform while crawling. A number of studies have shown that a cell undergoes rhythmic deformation by protruding and retracting the plasma membrane during migration. Membrane protrusions are formed either by (i) generation of actin networks mediated by the Arp2/3 complex, such as lamellipodia and invadopodia (for cancer cells); (ii) Arp2/3-independent extension of actin bundles, such as filopodia; or (iii) actin-free membrane blebs originating from intracellular hydrodynamic pressures (Schaks et al. 2019).

**Fig. 1:**
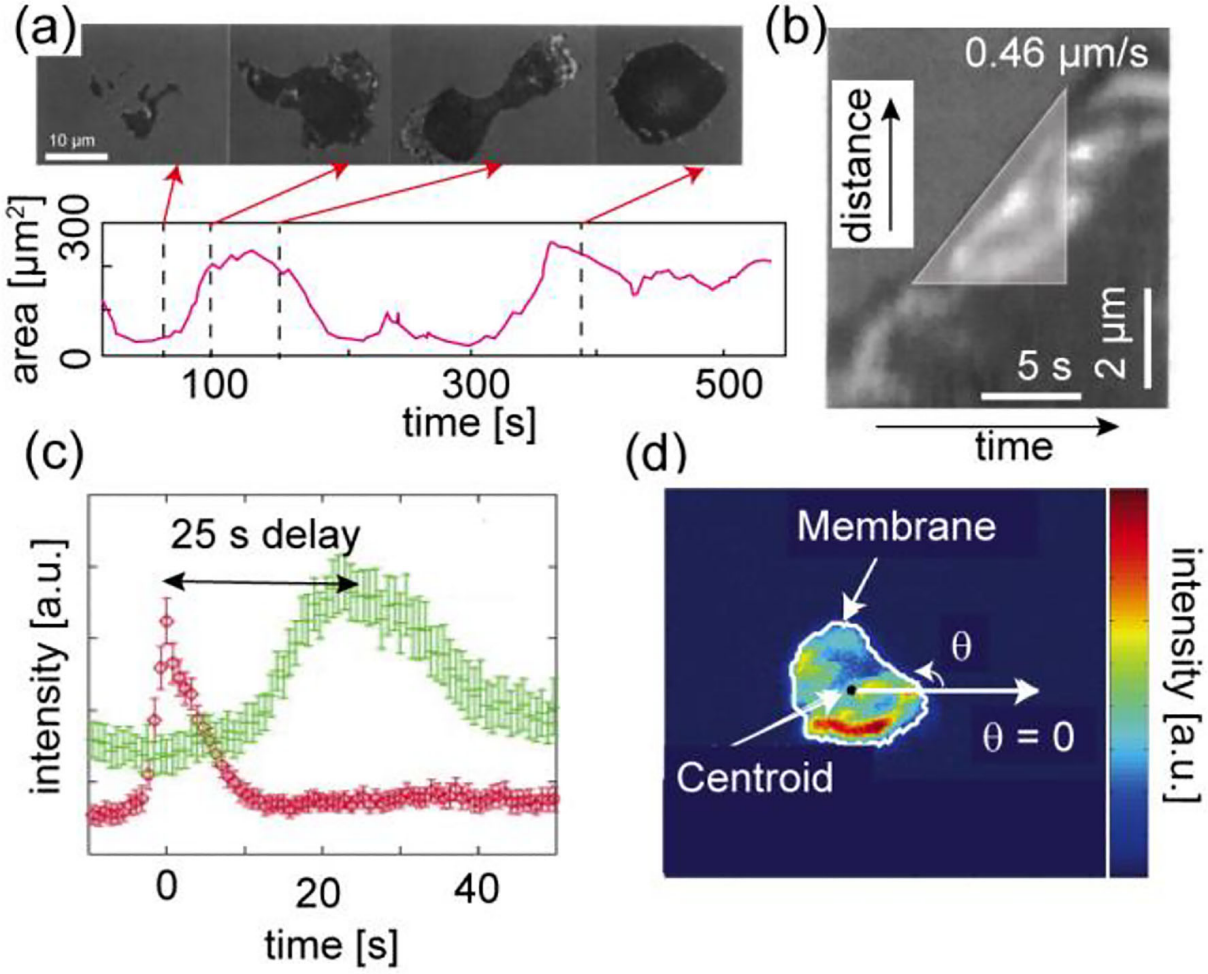
Spreading-contraction of membrane protrusions. (a) The spreading and contraction of adhesion contact of migrating *D. discoideum* on freshly cleaved mica, as imaged by microinterferometry. The region of the tight cell-substrate contact can be seen in dark gray because of destructive interference. (b) Kymogram of the leading edge plotted over time, yielding a spreading velocity of 0.46 μm/s. Figures adapted and modified from Schindl et al. (1995). (c) A solitary wave of actin labeled with RFP-LimEΔ (red) near the leading edge is followed by the delayed activation of the myosin II motor (green). Figure adapted and modified from Etzrodt et al. (2006). (d) Spatiotemporal distributions of actin, $Act\left(\theta, t\right)=\frac{\int {I}_{GFP}\left(\theta, t\right) rdr}{\int rdr}$ in migrating *D. discoideum* using GFP binding fusion proteins. Adapted from Maeda et al. (2008)

Figure 1a shows the microinterferometry images of a *Dictyostelium discoideum* (*D. discoideum*) migrating on freshly cleaved mica (Schindl et al. 1995). The shape deformation is characterized by periodic changes in adhesion contact areas to the substrates, indicating that a cell repeatedly undergoes the spreading-contraction cycle. As shown in Fig. 1b, the kymograms suggest that the velocity of a leading edge is constant during one spreading event, yielding *v* ≈ 0.46 μm/s. The protrusion and retraction of cell membranes also correlates tightly with the remodeling of underlying cytoskeletons (Clainche and Carlier 2008; Pollard and Borisy 2003). For example, the formation of crosslinked actin networks (actin “gel”) taking place at the front generates protruding forces in a rhythmic fashion (Yumura and Fukui 1985). Using the slime mold *D. discoideum*, Etzrodt et al. (2006) provided the evidence that a “solitary wave” of actin gel generated near the spreading front, labeled with RFP-LimEΔ, is followed by delayed myosin II activation (Fig. 1c) (Etzrodt et al. 2006). The advantage of simple and established cell lines enables the introduction of reporter systems. For example, by using the actin binding domain fused to GFP (Fig. 1d), Maeda et al. (2008) performed cross-correlation analysis and showed a strong correlation between actin condensation and membrane protrusion (Fig. 1e) (Maeda et al. 2008). Weiner et al. (2007) further showed that the propagation of the actin wave is regulated by reciprocal interactions between the actin-regulating complex and actin filaments bound to the cytoplasmic surface of cell membranes (Weiner et al. 2007). Here, binding of the actin-regulating complex to the membrane induces actin polymerization, while the produced actin filaments remove the complex from the membrane. Such reactions do not occur simply by mixing proteins in a liquid droplet. Many biochemical reactions are strictly confined to the proximity of membranes because membranes play vital roles in logistic control of various molecular processes during cell migration in a space- and time-dependent manner.

## Active roles of membranes: physical and biochemical views

What are the “switches” regulating actin gelation waves and spreading-contraction cycles? How is this process managed in space and time? As indicated in the previous section, proteins alone are not able to drive cell migration. Mounting evidence suggests that thermodynamic properties of lipids and their assemblies also play key roles in regulating cellular functions. Although there are many biochemical and biophysical studies that have focused on interactions between proteins, the role of lipids in regulating cellular functions has been widely overlooked.

To explain why many key biochemical reactions and signaling pathways are confined in the proximity of cell membranes, Hardt extended the classical Smoluchowski equation and calculated the mean diffusion time *τ* for collision in 2D and three-dimensional (3D) space (Hardt 1979):

$$ \left\langle {\tau}_{2D}\right\rangle =\frac{x^2}{2D}\ln \left(\frac{x}{r}\right)\;\mathrm{and}\;\left\langle {\tau}_{3D}\right\rangle =\frac{x^3}{3 Dr}. $$

*D* is the diffusion coefficient, *r* the radius of diffusing particles and *x* is the separation distance between two particles. The dependence of the mean diffusion time on the particle radius *r* is 〈*τ*_2*D*_〉 ∝  − ln(*r*) for 2D systems, whereas this dependence is 〈*τ*_3*D*_〉 ∝ *r*^−1^ in 3D systems. A clear influence of dimensionality on the relationship between *τ* and *r* indicates the energetic, and thus the “economic,” reason for many biochemical reactions being confined in quasi-2D space; i.e., “in” and “near” lipid membranes. Using cell-sized, water-in-oil droplets coated with lipid membranes, Yoshikawa and co-workers reported that the confinement of the reaction near the membrane accelerated gene expression (Kato et al. 2012). Remarkably, many proteins involved in cell migration are dissolved in the cytoplasm, remaining in a non-active, resting state. To activate their functions, these proteins first need to be recruited and bind lipids either by electrostatic binding to charged lipid head groups or by incorporation of hydrophobic moieties into the membrane core, which causes conformational changes to the proteins and subsequent activation. As described below, cell migration is a cellular process where lipids and their logistical self-assembly play major roles in regulating cellular functions.

**Fig. 2:**
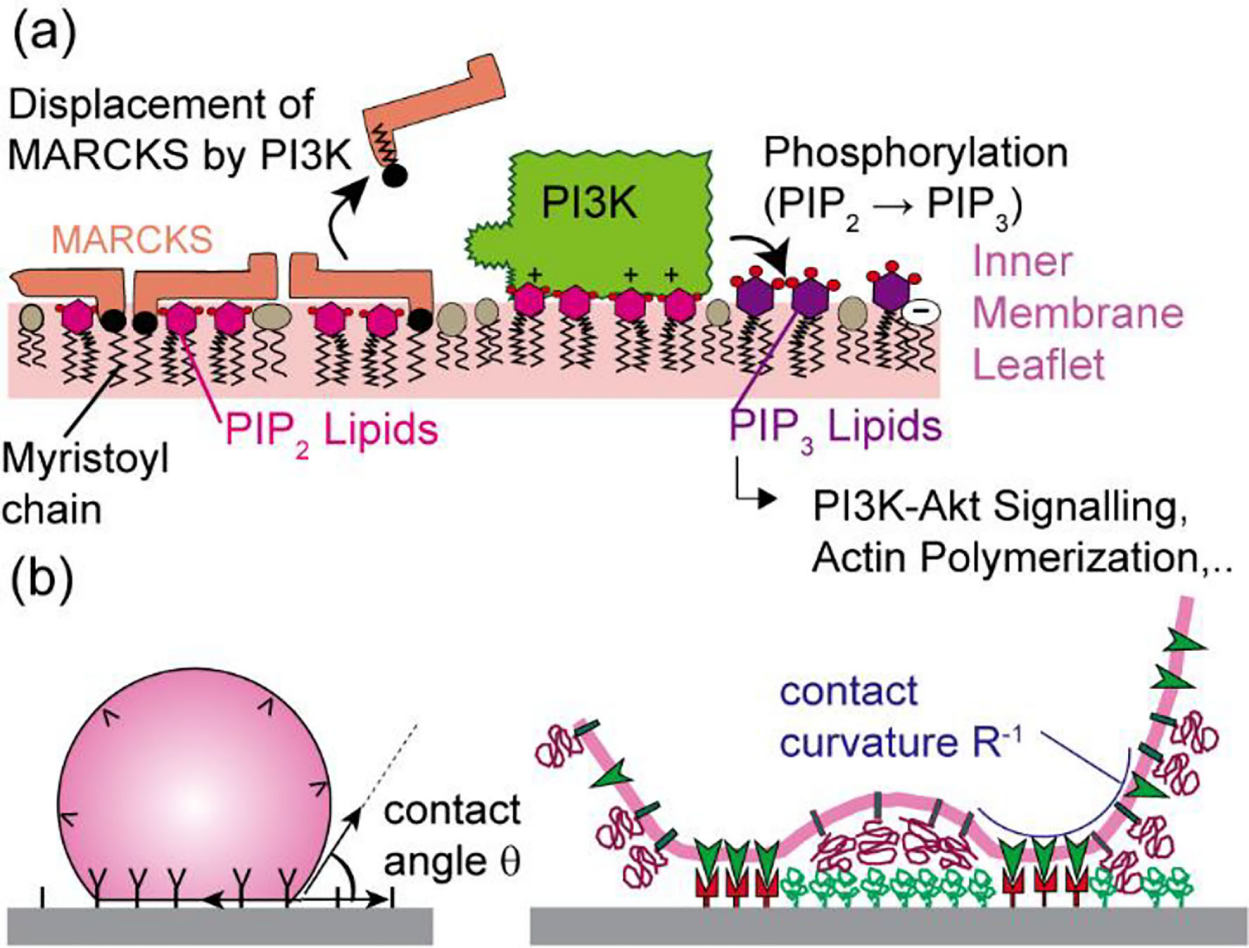
Plasma membranes as biochemical reaction centers. **a** Binding of activated phosphoinositide 3-kinase (PI3K) is initiated by phosphorylation of PIP2 to PIP3, which displaces the positively charged myristoylated alanine-rich C-kinase substrate (MARCKS). PI3K acts as a primary switch for many signaling processes because the phosphorylation product (PIP3) recruits various proteins to the cytoplasmic surface, such as Akt and Rho-GTPase. **b** The Bell, Dembo, and Bongrand model is based on osmotic pressure originating from attractive lock-and-key interactions under equilibrium (left), and membrane-mediated linker-linker attraction gives lateral phase separation (right)

Phosphoinositide 3-kinase (PI3K) is a primary membrane switch that triggers a wide variety of cellular processes, such as cell survival and cell migration (Fig. 2a). In the resting state, PI3K resides in the cytoplasm and remains inactive because binding to its substrate, phosphatidylinositol (4,5)-bisphosphate (PIP2), on the cytoplasmic membrane surface is blocked by the myristoylated alanine-rich C-kinase substrate (MARCKS). MARCKS binds to PIP2 by electrostatic attraction of 13 basic amino acids, and the myristoyl chain is incorporated into the hydrophobic membrane core (Aderem 1992; Wang et al. 2001). After activation with receptor tyrosine kinases, PI3K is recruited to the membrane surface and binds to PIP2 via phox-homology (PX) and C2 homology membrane interacting domains, and this binding event is driven by hydrophobic interactions (Chen et al. 2018; Scott et al. 2013). The activated PI3K displaces electrostatically bound MARCKS and phosphorylates PIP2 to PIP3, generating a “swarm” of PIP3 in the cytoplasmic leaflet of the membrane. Consequently, the self-assembled PIP3-enriched domains act as super affinity hotspots recruiting proteins that possess the highly specific pleckstrin homology (PH) domain, such as protein kinase B (Pilling et al. 2011).

Protein kinase B, which is often termed Akt, plays key roles in a variety of cellular processes, including metabolism, proliferation and migration (Hemmings and Restuccia 2012; Stambolic and Woodgett 2006). Transfer of Akt to/from lipid membranes spatiotemporally regulates the enzymatic functions of Akt. The activation of Akt upon binding PIP3 is a prerequisite for regulating cell proliferation, differentiation and migration. Conversely, permanent activation of the Akt signaling pathway causes overreactions in cells. The upregulation of Akt is a characteristic alteration found in various tumors, which suppresses apoptosis and promotes migration (Chin and Toker 2009). To avoid constant Akt upregulation, the level of PIP3 is lowered by PTEN, which dephosphorylates PIP3 to PIP2. On losing membrane affinity, Akt is released from the membrane and deactivated. Knockout of PTEN upregulates cell migration, whereas the dominant-negative Akt suppresses migration (Higuchi et al. 2001). Therefore, recruitment of Akt to the membrane by phosphorylation of PIP2 to PIP3 by PI3K, and release of Akt from the membrane by the dephosphorylation of PIP3 to PIP2 by PTEN are two competing pathways that regulate the activity of PI3K-Akt signaling, which are important upstream cell migration determinants (Seo et al. 2014).

## Adhesion domains in membranes as biochemical reaction centers

Different from bacteria swimming by rotational motions of flagella, “crawling” cells grip the extracellular matrix or neighboring cells by focal adhesion contacts and actively generate forces.

Bell et al. (1984) adapted the classical Young-Dupré equation and theoretically described cell adhesion as the manifestation of wetting and osmotic pressure, originating from the attractive lock-and-key interactions under thermodynamic equilibrium (Bell et al. 1984), Π = *γ*(1 − cos *θ*), where *γ* is the membrane tension and *θ* the contact angle defined in Fig. 2b (left). Note that the Young-Dupré equation for a classical Newtonian liquid must be corrected to deal with cell adhesion because cell membranes deform not only plastically but also elastically (Bruinsma 1995; Bruinsma et al. 2000; Purrucker et al. 2007). Cell adhesion is mechanically controlled by the interplay between attractive, short-range forces between specific ligand-receptor pairs (characteristic distance ≈ 15 nm) and repulsive, medium-range forces (characteristic distance ≈ 30 nm) generated by glycocalyx (Bruinsma and Sackmann 2001; Sackmann and Smith 2014). For the quantitative determination of adhesion strength and dynamics of cells, it is necessary to design surrogate substrates with well-defined ligand identity and arrangement. Planar lipid membranes functionalized with transmembrane cell receptors and recombinant proteins, called “supported membranes”, can offer unique advantages because controlled self-assembly of proteins enables the precise control of the lateral distance (hence density) of adhesion ligands at nanometer accuracy (Groves and Dustin 2003; Sackmann 1996; Tanaka and Sackmann 2005). Microinterferometry, called reflection interference contrast microscopy (RICM), is a powerful tool to visualize changes in shape and size of adhesion contacts as well as the dynamic phase separation caused by adhesion (Bruinsma et al. 2000; Burk et al. 2015; Goennenwein et al. 2003; Kaindl et al. 2012; Monzel et al. 2018). In situations where the osmotic pressure is smaller than the van der Waals energy per unit area, *Π*_rep_ < *W*_vdw_, it is energetically favorable to exclude repellers from the tight adhesion zone (Fig. 2b, right). Thus, adhesion is inevitably accompanied by lateral phase separation leading to the formation of adhesion domains, whose spatial organization is determined by the persistence length of membrane deformation, *ξ* ≈ 50 nm (Lipowsky and Sackmann 1995). By treating a lipid membrane as the surface of a fictitious fluid, Bruinsma and Sackmann (2001) described the transition from weak to strong adhesion as a first order de-wetting transition (Bruinsma and Sackmann 2001).

## Adhesion domains near the spreading front regulate actin polymerization

**Fig. 3:**
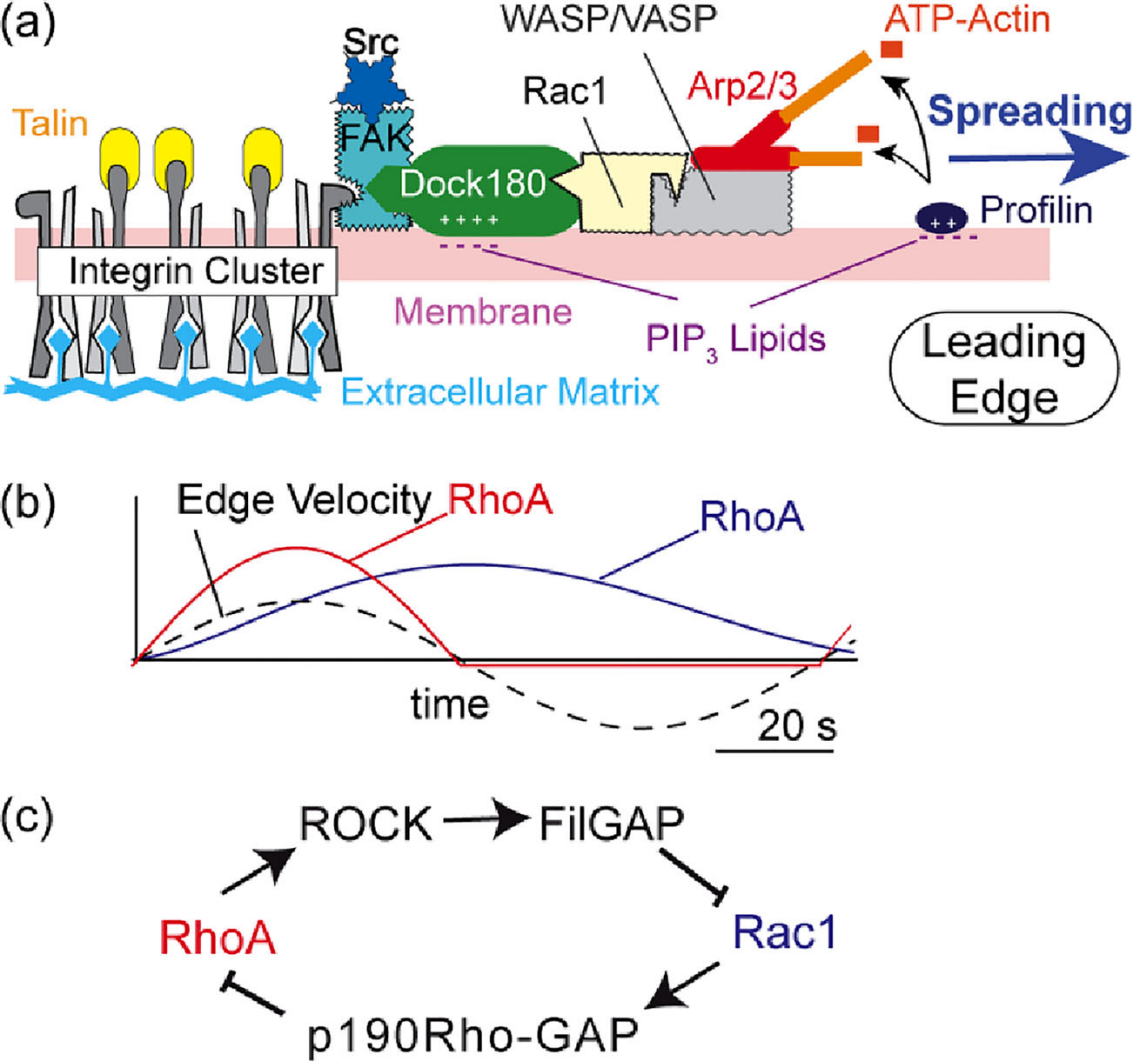
Coordination of adhesion domains, Rho GTPase, and cytoskeletons. **a** Polymerization of actin filaments near the leading edge. Clusters of integrin (adhesion domain) activate focal adhesion kinase (FAK) and tyrosine-protein kinase (Src), which recruits the PI3K switch to the cytoplasmic surface. The binding of Dock180 to PIP3 on the membrane surface activates Rac1, and the binding of Rac1 to WASP/VASP activates Arp2/3 gelator and hence promotes actin gelation near the spreading front. **b** Spatiotemporal coordination of Rho GTPase (RhoA and Rac) during periodic spreading and contraction cycles. The emergence of oscillating patterns indicates the presence of at least one negative feedback. **c** Molecular mechanism of the antagonistic interplay of RhoA and Rac1

Integrin, a heterodimeric transmembrane receptor, is one of the most important players mediating interactions between the extracellular matrix and the actin cytoskeleton (Huttenlocher and Horwitz 2011; Hynes 2002). During cell migration, integrin clusters recruit different adaptor proteins, such as talin, kindlin, vinculin, and tensin. These adaptor proteins anchor actin cytoskeletons to the cytoplasmic surface of cell membranes (Fig. 3a). In particular, the binding of talin to the cytoplasmic domain of the integrin β subunit elevates the affinity of integrin to its ligand, which is highly important in development and diseases (Wegener et al. 2007). The formation of integrin clusters in the membrane near the leading edge is followed by autophosphorylation of focal adhesion kinase (FAK), which is a protein kinase associated with focal adhesion contacts. Activated FAK acts as a substrate for tyrosine-protein kinase (Src) (Oudart et al. 2016; Sulzmaier et al. 2014) and hence the binding of FAK and Src results in the activation of both kinases. As shown in Fig. 3a, adhesion promotes the binding of PI3K to the activated FAK/Src at its major autophosphorylation site Y397.

Actin polymerization near the leading edge requires the feed of polymerizable actin monomers bound to ATP (Clainche and Carlier 2008). Free, non-active monomers are available either from the β-thymosin-bound actin buffer or from the pool of non-active (ADP-bound) monomers cleaved by cofilin. Cofilin is deactivated by phosphorylation of Ser-3 by LIM kinase (Arber et al. 1998) and reactivated by cofilin-phosphatase slingshot dephosphorylation (Nishita et al. 2004). Profilin frees non-active actin monomers from sequester proteins and phosphorylates these monomers to provide active, ATP-bound monomers. Profilin is inactivated by electrostatic interaction with negatively charged PIP2/PIP3, which is enriched near adhesion domains (Senju et al. 2017). Because polymerization is dependent on the delivery of monomers to the spreading front, the periodicity of the spreading-contraction cycle is regulated by the lifetime of PI3K activation. Actin polymerization correlates with a transient increase in PIP3 levels in many cell types, which further suggests the influence of PI3K on cell migration (Rickert et al. 2000).

Rho family small guanosine triphosphate-binding proteins (Rho-GTPases) are key switches contributing to the formation of lamellipodia and filopodia formation in migrating cells. They are active when bound to GTP and inactive when bound to GDP. Rho-GTPases are activated by guanine nucleotide exchange factors (GEFs) and inactivated by GTPase activating proteins (GAPs). Many GEFs for Rho family GTPases have Dbl homology (DH) binding to both the catalytic guanine-nucleotide exchange domain and PH domain that bind to PIP3 (Lemmon and Ferguson 2000). Note that recruitment of Rac1 to the plasma membrane surface is necessary for activation because Rac1 residing in the cytoplasm is in the resting state and hence inactive.

As shown in Fig. 3a, thrust force generated by actin polymerization near the leading edge is driven by the recruitment of activated Rac1 to newly formed adhesion domains in plasma membranes. Among the GEF family, dedicator of cytokinesis (Dock180) is a non-typical GEF because it cannot act alone as a GEF (Brugnera et al. 2002). Dock180 first binds to PIP3 expressed on the cytoplasmic surface of a plasma membrane (Vermeren et al. 2010), and complex formation with engulfment and cell motility protein (ELMO) enables coupling to the substrate Rac1 (Brugnera et al. 2002; Katoh and Negishi 2003; Patel et al. 2011). Rac1 recruits and activates the Wiskott-Aldrich syndrome protein (WASP), which promotes actin polymerization mediated by Arp2/3. The Rac1-WASP complex also interacts with the vasodilator-stimulated phosphoprotein (VASP) and promotes actin polymerization (Havrylenko et al. 2015; Ridley 2015). The binding of Rac1 to the WASP/VASP promoter is necessary for the activation of actin gelator Arp2/3 because the basic activity of Arp2/3 is low. Similar to PI3K, Dock180 binds electrostatically to PIP3 via a polybasic C2 domain (Premkumar et al. 2010). Modulating the coupling of Dock180 with lipid membranes by PI3K provides strong positive feedback between the activation of Rac1 and PI3K.

The actin polymerization machinery is also sensitive to exogenous forces. Dock180, the activator of Rac1, is connected to the strain-sensitive Crk/Cas complex coupled to FAK/Src adjacent to integrin clusters, whereas RhoA is kept inactive during this period by the specific inhibitor p190Rho-GAP (Nimnual et al. 2003). This force-generating period is terminated by deactivation of Rac1 by its specific inhibitor, filamin A (FLNa)-binding GAP (FilGAP), which binds to force-sensitive crosslinker filamin A and suppresses actin polymerization near the leading edge (Ehrlicher et al. 2011; Ohta et al. 2006; Rognoni et al. 2012). The activation of FilGAP requires phosphorylation by Rho kinase (ROCK), which is a downstream effector of RhoA. ROCK also activates LIM kinase and is therefore downstream of cofilin. In contrast, cofilin is inhibited in regions where the levels of RhoA are high (Maekawa et al. 1999). As a consequence, RhoA and ROCK stimulate myosin II and PTEN, which promotes retraction (Li et al. 2005). After a while, RhoA is switched off again by binding of p190Rho-GAP, which triggers the next force generation cycle.

Notably, other adhesion molecules utilize this actin polymerization machinery mentioned above. For instance, glycoprotein CD44 is another important adhesion molecule that monitors changes in the extracellular matrix and adapts the growth, survival and differentiation of cells (Ponta et al. 2003). Interactions of CD44 with matrix glycosaminoglycan hyaluronan (HA) are influenced by glycosylation of the extracellular domain, clustering of CD44 and phosphorylation of the cytoplasmic domain of CD44. Because CD44 molecules are localized near the spreading front, it has been suggested that the change in CD44-HA interactions modulates the migration of cells significantly in the extracellular matrix enriched with HA. The cytoplasmic domain of CD44 is coupled to actin cytoskeletons by the Band 4.1 superfamily, the ERM (ezrin, radixin and moesin) protein. As adhesion mediated via CD44-HA binding activates Rac1, activated Rac1 attracts the WASP/VASP promoter for the formation of Arp2/3-mediated polymerization of actin, following a similar scenario to the one described above (Bourguignon et al. 2007; Oliferenko et al. 2000).

## Antagonistic interplays of GTPases on membrane surfaces

Mounting evidence suggests that the rhythmic spreading of the leading edge is coordinated by the interplay of Rho GTPases, such as Rac1 and RhoA (Kraynov et al. 2000; Kurokawa and Matsuda 2005; Nalbant et al. 2004; Pertz et al. 2006). Machacek et al. (2009) monitored spatiotemporal coordination of Rac1 and RhoA activity in the proximity of the leading edge of a migrating *Dictyostelium* (Machacek et al. 2009). As shown in Fig. 3b, the activation of RhoA and the spreading of the leading edge occur simultaneously with no phase delay, while the increase in the Rac1 level follows subsequently. RhoA activates mDia, which is associated with membrane protrusions (Kurokawa and Matsuda 2005). Palazzo et al. (2001) demonstrated that mDia, but not ROCK, is a RhoA downstream effector involved in microtubule organization in the proximity of the leading edge (Palazzo et al. 2001). Moreover, the breakdown of stable, RhoA-mediated microtubules near the basal membrane is a key step in epithelial-mesenchymal transition, a key process of development and pathogenesis (Nakaya et al. 2008). As presented in Fig. 3b, the activation of RhoA and Rac1 exhibited a clear phase shift, indicating that these two proteins are not tightly coupled to the spreading front both in space and time. In fact, the activation of Rac1 peaks at 1.8 μm behind the leading edge with a delay of ≈ 40 s (Machacek et al. 2009). The activation levels of Rac1 remain high even when the retraction of membrane protrusions starts. The “tail” of the lower level of Rac1 activation is present even at the beginning of the next cycle of membrane protrusion. Notably, the location of Rac1 activation, 1.8 μm behind the leading edge, coincides with the location of maturating adhesion contacts (Zaidel-Bar et al. 2003). This indicates that Rac1 stabilizes membrane protrusions by reinforcing adhesion sites to balance membrane spreading initiated by RhoA.

From a mathematical viewpoint, the oscillatory activation of RhoA and Rac1 suggests the involvement of a mutual antagonism (Fig. 3c) because the emergence of stable oscillatory patterns generally requires at least one negative feedback loop (Nguyen 2012; Pigolotti et al. 2007). Therefore, the logistic recruitment of an activator (GEF) and an ingibitor (GAP) to the membrane domains is necessary for the spatiotemporal regulation of periodic membrane protrusions (Fig. 1). The periodicity of the deformation seems different between cell types. The slime mold *D. discoideum* exhibits an excitable deformation every 2–4 min (Li et al. 2008; Maeda et al. 2008), whereas human hematopoietic stem cells showed a periodic deformation every 5 min (Ohta et al. 2018). Because the adhesion contacts act as the reaction center for actin remodeling, the highest deformation rate coincides with the lifetime of new adhesion domains. For example, *D. discoideum* followed the change in chemotactic gradients up to a rate of 0.02 Hz (Meier et al. 2011) and human hematopoietic stem cells undergo periodic deformation with a frequency of 0.03 Hz (Ohta et al. 2018). Intriguingly, the active deformation of cancer cells, such as murine pancreatic cancer cells and human gastric cancer cells, exhibit no periodic patterns during migration (Kaindl et al. 2012), suggesting the continuous elevation of Rac1 activation. This seems reasonable because previous accounts reported the overexpression of Rac1 in human patient samples of breast, gastric, testicular, oral squamous cell, lung, and pancreatic cancers (Heid et al. 2011; Karlsson et al. 2009). The activation of Rac1 by PI3K has also been reported to play critical roles in tumorigenesis in the murine pancreas (Wu et al. 2014).

## Retraction of stress fibers near the trailing end

**Fig. 4:**
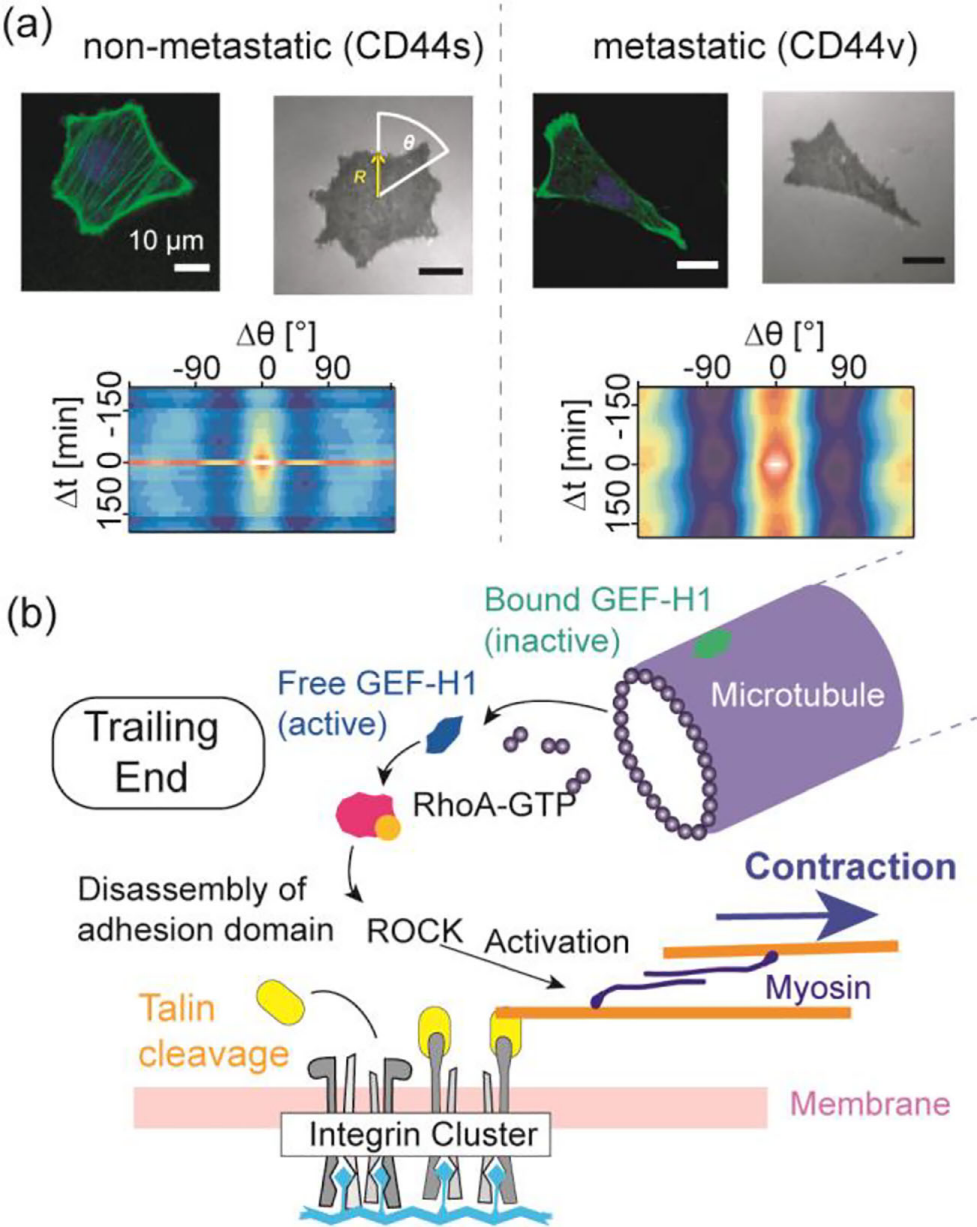
Spreading/retraction balance affects morphology and migration. (a) The balance of spreading and retraction determines the cell morphology. The autocorrelation function of non-metastatic murine pancreatic cancer cells expressing endogenous CD44s implies that a hexagonal cell undergoes a rotational motion. The corresponding data of cells expressing the variant exon-containing isoform (CD44v) suggests that a linearly stretched cell undergoes directional migration persistently. The different morphology and mode of migration can be attributed to the accessibility of a protease enzymatically cleaving the extracellular domain of CD44. (b) The molecular mechanism of retraction of the trailing edge. Proteolytic degradation of talin by calpain disconnects integrin from actin and disassembly of adhesion domains near the trailing end. The activation of RhoA by microtubule-mediated GEF (GEF-H1) stimulates myosin II and PTEN, resulting in the disruption of weak adhesions by contraction

The balance between the speed of spreading at the leading edge and that of retraction at the trailing end determines the cell shape and mode of mesenchymal migration (Lauffenburger and Horwitz 1996). For example, the spreading of a fibroblast at the front is faster than the retraction at the rear, which results in a triangular shape. In contrast, a migrating keratinocyte takes a crescent shape because the speed of spreading and that of retraction are comparable. Previously, Kaindl et al. (2012) compared the morphological dynamics and migration patterns of metastatic and non-metastatic pancreatic cancer cells on HA-coated surfaces (Kaindl et al. 2012). As presented in Fig. 4a, non-metastatic cells expressing endogenous CD44 exhibited an isotropic expression of stress fibers in the periphery. Intriguingly, the autocorrelation function *Γ*(*θ*,*t*) of shape deformation $\varGamma \left(\theta, t\right)=\frac{\left\langle R\left(\theta +\Delta \theta, t+\Delta t\right)\bullet R\left(\theta, t\right)\right\rangle }{\left\langle {\left[R\left(\theta, t\right)\right]}^2\right\rangle }$ exhibits three axes of rotational symmetry that quickly decay over time, suggesting that the cell adopts a hexagonal morphology and undergoes a spinning motion. In contrast, metastatic cells expressing the variant exon-containing isoform (CD44v) show a clear front-rear asymmetry, implying that the spreading of the leading edge is faster than the retraction of the trailing end. The calculated autocorrelation function is characterized by two symmetry axes that persist over time, indicating that the cell is linearly stretched and undergoes directional migration. The difference in the shape and migration phenotypes is attributed to the change inserted in the extracellular domain of CD44v, which increase access of a matrix metalloprotease and thus the efficiency of retraction (Kaindl et al. 2012).

The retraction of the trailing end is driven by two mechanisms (Fig. 4b): (i) proteolytic degradation of matured adhesion domains; and (ii) disruption of weak adhesion contacts by actin stress fibers and microtubules. In the first mechanism, calpain induces the proteolysis of talin, which disrupts the links between the integrin β_1_ subunit and actin. The calpain activity is suppressed near the leading edge because calmodulin suppresses the proteolytic activity of calpain near the leading edge by keeping the local concentration of Ca^2+^ ions below the activation level. Conversely, calpain near the trailing end is activated by the elevation of Ca^2+^ concentration by the transient receptor potential melastatin-related 7 (TRPM7) channel in adhesion domains (Su et al. 2006) and the Fam 38 (PIEZO1) channel in endoplasmic reticulum (McHugh et al. 2012). Franco et al. (2004) showed that the proteolytic cleavage of talin by calpain significantly influences the disassembly of other proteins in adhesion domains, suggesting that the dissolution of adhesion domains causes adhesion turnover (Franco et al. 2004). In particular, talin seems to play a key role in the maintenance of focal adhesion contacts, as a genetic knockdown of talin reduced adhesion free energy and the membrane tension of *D. discoideum* (Simson et al. 1998). Proteolytic cleavage of the integrin-actin binding does not reduce the cell adhesion but also promote the cell migration . In fact, the upregulation of Fam38 and TRPM7 substantially facilitates the migration and invasion of cancer cells (Lefebvre et al. 2020; McHugh et al. 2012). In the second mechanism, the key force generator disrupting the weak adhesion contacts is stress fibers (Yumura and Fukui 1985). Microtubules activate RhoA via microtubule-associated GEF, called GEF-H1 (Guilluy et al. 2011). GEF-H1 bound to a microtubule is inactive, but GEF-H1 released from a depolymerizing microtubule is active. Free GEF-H1 activates RhoA, which stimulates the effector ROCK. This is followed by the phosphorylation of its downstream targets such as PTEN and myosin light chains, resulting in the retraction of stress fibers (Li et al. 2005). As described above, the activation of PTEN antagonizes PI3K function and thus downregulates PIP3 levels. The targeting of focal adhesion contacts by the plus ends of microtubules has been demonstrated by total internal reflection fluorescence microscopy (Krylyshkina et al. 2003). The growing microtubules “patrol” in the vicinity (≈ 50 nm) of the dorsal membrane surface towards the adhesion domains near the leading edge, but move away from the membrane surface near the trailing end during retraction.

Note that all key molecules involved in these competing pathways, GTPases, GEFs, and GAPs, are activated only on the membrane surface. Therefore, the confinement of all the above-mentioned reactions in quasi-2D space is an effective strategy because diffusion in 2D is much less dependent of the molecular size compared with diffusion in 3D bulk. This makes the distance that molecules need to diffuse to undergo these reactions shorter than the cell size. The physical consideration of 2D diffusion enables us to explain the simultaneous formation of broad lamellipodia by Rac1 and fingerlike filopodia by RhoA in migrating cells. Taking data from various experiments from the First World Cell Race, Maiuri et al. (2012) suggested the presence of a universal law between deformation and motion (Maiuri et al. 2012). The analysis of migration trajectories on 1D adhesive tracks suggested an exponential correlation between migration speed *v* and persistence time *τ* of cells, *τ*~*e*^*λv*^, which originates from the transport of polarization factors by the retrograde flow of actin (Maiuri et al. 2015).

## Membrane-localized reactions guide cell polarization

From a biochemical viewpoint, global polarization at the cellular level can be characterized by a non-uniform disution of cytoplasmic signaling molecules and an asymmetric organization of cytoskeletal proteins. From a biophysical viewpoint, polarization at the cellular level can be characterized by shape asymmetry, non-uniform expression of cell adhesion molecules and hence non-uniform frictional coupling to contact surfaces and an asymmetric orientation order of cytoskeletons.

**Fig. 5:**
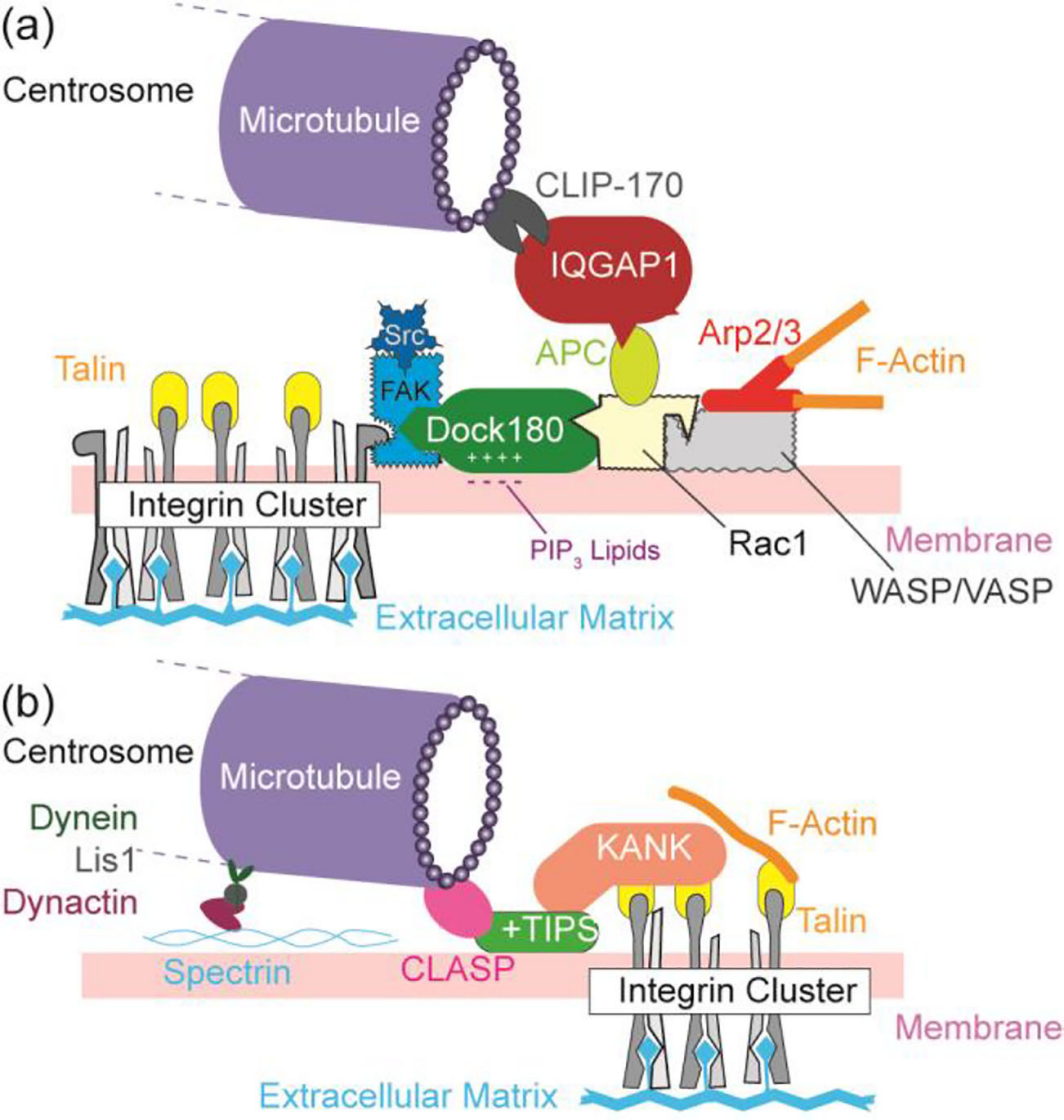
Cell polarization regulated by harnessing microtubules to membranes. Crosslinking of actin filaments and microtubules near the leading edge by (a) the IQGAP1-CLIP-170 complex and (b) KANK-CLASP complex. Note that the IQGAP1-APC-Rac1 complex is stable only near the leading edge, where [Ca^2+^] is sustained at a low level by calmodulin

By examining at shorter length scales inside cells, the axis of intracellular polarization is defined by the nuclear-centrosome axis (Luxton and Gundersen 2011), which clearly indicates that microtubules critically determine the stability of cell polarization and hence the persistence of cell migration. A prerequisite for the establishment of a stable centrosome-nuclear axis is the recruitment and tethering of the plus end of the microtubule to the plasma membrane close to the leading edge (Etienne-Manneville 2013). Among Rho GTPase regulating the dynamic organization of actin filaments and microtubules, Rac1 regulates the location and activity of the effector protein, IQGAP1 (Briggs and Sacks 2003b; Kuroda et al. 1996). To form an array of polarized microtubules connected to the cell cortex, the plus end of microtubules needs to be stabilized by plus-end-binding proteins (+TIPS), such as CLIP-170, EB1, CLASP, and actin crosslinking factor 7 (Acf7) (Gundersen 2002; Schuyler and Pellman 2001). Because IQGAP1 selectively binds to CLIP-170 on the plus end of microtubules (Fukata et al. 2002), the IQGAP1-CLIP-170 complex and hence the plus end of microtubules is recruited to Rac1. As IQGAP1 directly interacts with the adenomatous polyposis coil (APC) and forms a triplex with activated Rac1 (Watanabe et al. 2004), IQGAP1 serves as a cross-linker that connects microtubules and actin filaments (Fig. 5a). The plus end of microtubules is also recruited by the binding of Acf7 to the Dock180-ELMO-Rac1 complex that increases the persistence of membrane protrusions (Margaron et al. 2013). As described in the previous section, Rac1 is localized near maturating adhesion contacts in plasma membranes, which are about 1–2 μm behind the leading edge (Zaidel-Bar et al. 2003). Near actin-microtubule junctions, stathmin, activated by Rac1, stabilizes microtubules. The binding of IQGAP1 to calmodulin keeps the local Ca^2+^ ion concentration near the leading edge low, which is a prerequisite for the stabilization of the IQGAP1-APC-Rac1 complex (Briggs and Sacks 2003a). This enables this stable triplex to localize cortex microtubules near the leading edge via binding of CLIP-170 to IQGAP1. An additional key factor stabilizing the nuclear-centrosome polarity is the Par polarity complex, which consists of Par-3/Par-6/aPKC (atypical protein kinase C) (Joberty et al. 2000; Lin et al. 2000). In migrating cells, the Par complex is enriched near the spreading front through the directed flow of actin. The Par complex, activated by Cdc42, inhibits RhoA and activates Rac1. Because the activated Par complex binds to the plus end of microtubules, the connection of microtubules and the cell cortex contributes to the positioning of centrosomes and hence the stabilization of front-rear asymmetry (Peglion and Goehring 2019).

Bouchet et al. (2016) reported that the forward movement of the centrosome is mediated by KANK1, which crosslinks microtubules to focal adhesion contacts (Bouchet et al. 2016). KANK1 selectively binds to the rod-like domain of talin and recruits the stabilization sites of the plus end of cortical microtubules, such as CLASP. Because talin in focal adhesion contacts near the leading edge is not fully occupied by actin filaments, KANK1 serves as an adaptor that crosslinks the CLASP-microtubule and talin in focal adhesion contacts (Fig. 5b). Conversely, adhesion contacts near the center are fully coupled to actin filaments. Sun et al. (2016) showed that KANK2 diminishes talin-actin binding near the center, induces the sliding of integrin-ligand binding and reduces the migration velocity (Sun et al. 2016). Intriguingly, the depletion of CLASP does not affect KANK2-induced sliding, indicating that the KANK2-talin interaction near the center does not involve microtubules. More recently, Rafiq et al. (2019) showed that manipulation of KANK caused the decoupling of microtubules from adhesion contacts (Rafiq et al. 2019). These data gave supporting evidence that adhesion domains in cell membranes near the leading edge spatiotemporally coordinate the directed movement of the centrosome and hence the polarity of the nuclear-centrosome axis in migrating cells. The position of the centrosome is controlled by dynein anchored to spectrin on the cytoplasmic membrane surface (Kardon and Vale 2009; Reck-Peterson et al. 2018). The membrane-anchored dynein exerts force on the microtubule and pulls the centrosome by using Lis1 (Fig. 5b) (Smith et al. 2000). Lis1 Lis1 promotes the formation of an active complex with dynactin (Elshenawy et al. 2020) and serves as a molecular “clutch”, stabilizing the dynein-microtubule attachment (Huang et al. 2012). Intriguingly, inhibition of actomyosin did not affect cell migration in soft, 3D environments (Rhee et al. 2010), suggesting that migration of cells under low tension conditions is regulated by dynein.

## Theoretical models of membrane protrusion and migration

After the 1D model of persistently migrating cells by DiMilla et al. (1991) (DiMilla et al. 1991), several theoretical approaches have been developed to model cell dynamics and the underlying mechanisms (Aranson 2016). The stochastic model equations were proposed for the center of mass or polarity vector of a migrating *D. discoideum* in vegetative and starved states (Li et al. 2008; Takagi et al. 2008), but these models did not include the role of active deformation or membrane protrusions. Theories of active gel have also been applied as a 1D model of cell migration, describing the retrograde flow of actin flow and propulsion of cells (Carlsson 2011; Kruse et al. 2006). Unfortunately, however, these models are currently unable to handle the shape deformation and hence membrane protrusions.

From this context, the phase field approach is a promising strategy to represent cell migration driven by membrane deformation (protrusion) caused by chemical reactions inside the cell (Camley et al. 2017; Taniguchi et al. 2013). Within this framework, the cell membrane is an interface subjected to tension, and a force balance between the spreading front and retracting end determines the cell shape. The direction of migration is regulated by chemical processes modeled by reaction-diffusion equations. For example, Taniguchi et al. (2013) used the phase map analysis of PIP3 waves and demonstrated that the deformation of *D. discoideum* can be characterized by the number, topology and position of organizing centers of rotating chemical waves, called phase singularities (Taniguchi et al. 2013). These approaches are able to couple the chemical reaction inside cells and deformation and motion of cells to some extent. Camley et al. (2017) modeled how the combination of membrane tension and chemical polarity, corresponding to the Rho GTPase-driven actin polymerization at the spreading front, regulates shape, migration speed and migration patterns (Camley et al. 2017). However, these models still do not account for the degrees of freedom of adhesion or the confinement of the reaction near the membranes. Ziebert and Aranson proposed a 2D phase field model including the degrees of freedom for adhesion (Ziebert and Aranson 2013; Ziebert and Aranson 2014), and proposed a more generalized minimal model describing a crawling cell in 3D (Tjhung et al. 2015). Giese et al. (2015) simulated the polarization of yeast *Saccharomyces cerevisiae*, where they introduced the influence of membranes by taking Rho GTPases in an active membrane-bound state and an inactive cytosolic state (Giese et al. 2015). Here, the membrane was modeled as a thin layer that allowed lateral diffusion, whereas the cytosol was a closed compartment with a finite volume. The simulations could recapitulate the influence of size (volume), protrusion (local curvature) and membrane inhomogeneity. Although this seems to be an interesting strategy, the shape was introduced merely as a static feature restricting molecular aggregation.

**Fig. 6:**
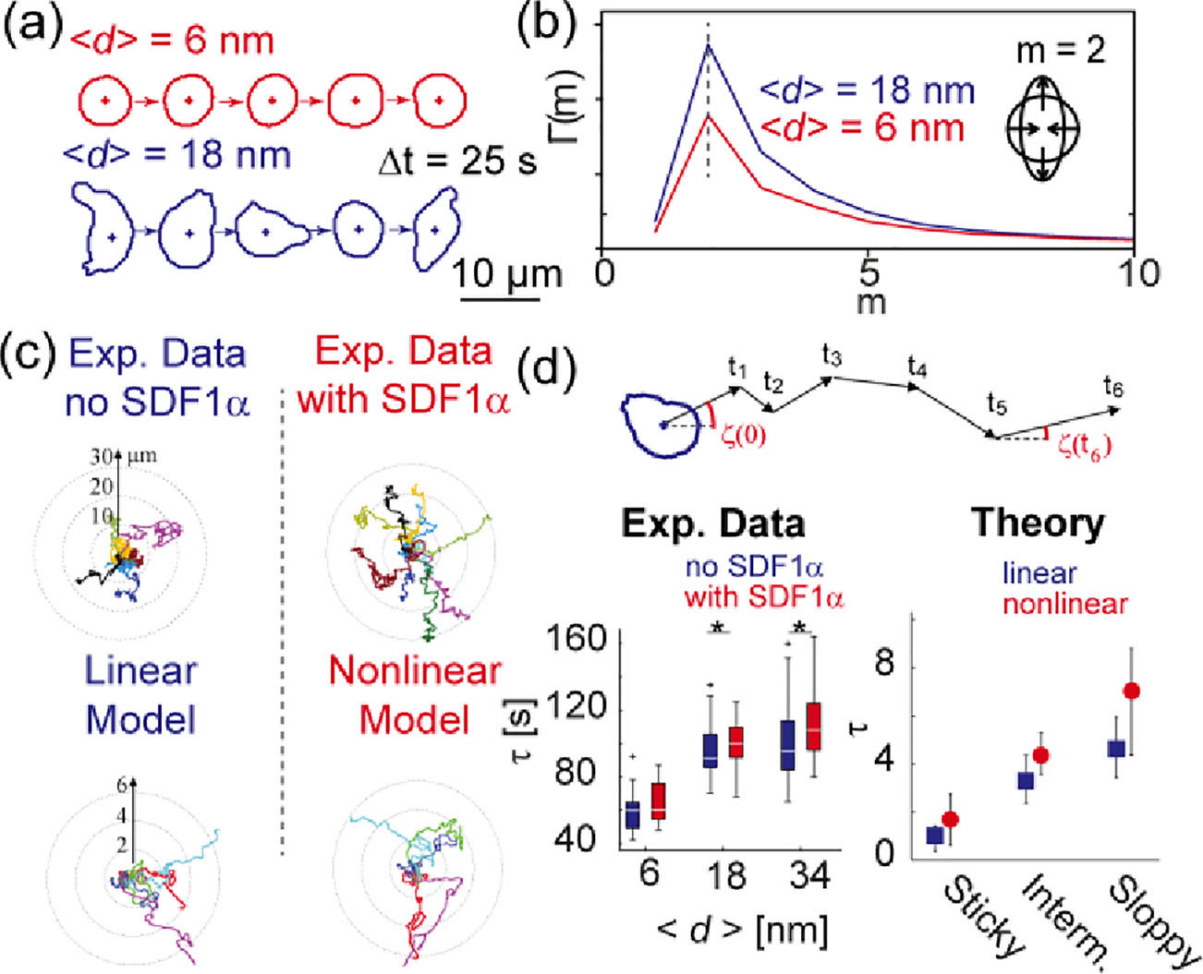
Quantitative theoretical modeling of migrating cells. Use of precisely functionalized supported membranes enables the quantitative comparison of theoretical calculations and experimental data. (a) Dynamic deformation of human hematopoietic stem cells on substrates displaying ligand molecules (SDF1α) at intermolecular distances of <*d*> = 6 and 18 nm. (b) Power spectrum calculated for the *m*th mode, indicating that hematopoietic stem cells undergo elliptic deformation (*m* = 2) in a <*d*> dependent manner. (c) Direct comparison of experimentally determined migration trajectories and simulations. Data in the presence of a physiological chemokine [SDF1α] = 5 ng/mL is only reproduced with the nonlinear model. Note that 2 in the simulation coincides with 10 μm as the normalization of the simulation space was normalized by the cell radius (5 μm). (d) Comparison of the correlation time between experiments and simulations obtained by $c(t)=\left\langle \cos \zeta (t)\cos \zeta (0)+\sin \zeta (t)\sin \zeta (0)\right\rangle \sim {e}^{-\frac{t}{\tau }}$. Because time was normalized by the periodicity of oscillatory deformations (30 s), *τ* = 2 in the simulation corresponds to 60 s

Ohta and coworkers applied the equation of motion for a deformable, self-propelled particle (Ohta and Ohkuma 2009) that describes the membrane protrusion and motion of crawling cells undergoing active deformation by excitable (Ohta et al. 2016) and periodic forces (Ohta et al. 2018). The center of mass velocity *v* is given by *v* = 2|*γ*|*s*_2_*s*_3_, where *γ* is the mobility, and *s*_2_ and *s*_3_ are principal deformation tensors. Here, the first derivative of the *m*th deformation tensor with respect to time is represented by the combination of the relaxation rate *κ*_m_, periodic active deformation force *g*_m_ with noise *ξ*_m_, and the nonlinear coupling term between deformation and velocity *b*_m_. This model was recently applied to simulate the active deformation and motion of primary human hematopoietic stem cells from donors, which were recorded by label-free live cell imaging (Ohta et al. 2018). Here, by using quantitatively functionalized supported membrane constructs as the model of bone marrow microenvironments (Tanaka and Sackmann 2005), the mobility *γ* can be controlled by precisely adjusting the intermolecular distance between ligand molecules on the surface <*d*> at nanometer accuracy. Intriguingly, the mobility *γ* and hence <*d*> significantly affected the active deformation (Fig. 6a). Here, the energy dissipation resulting from active deformation can be calculated from the summation of the power spectrum from the *m*th mode deformation, $\sum \hat{\Gamma_{\mathrm{m}}}$, which coincides with the sum of the relaxation rates, ∑*κ*_m_ (Fig. 6b). The migration trajectories calculated using this model could successfully reproduce the experimental migration trajectories of human hematopoietic stem cells on substrates with different <*d*>. Moreover, the function of the chemokine in bone marrow (SDF1α) was well represented as the nonlinear coupling between the deformation and motion (Fig. 6c). The main advantage of such a simple physical model enables direct, quantitative comparison of simulations with the corresponding experimental data obtained from the spatiotemporal imaging of label-free live cell imaging (Fig. 6d). Thus, this approach is suited for modeling the dynamics of primary cells, such as those from human donors. In stark contrast to the phase field model, this model does not include chemical reactions and signaling pathways inside cells. Therefore, combination of the phase field-type approaches and shape-motion simulations seem to be a promising strategy for the theoretical modeling of cell migration, ranging from established cell lines with reporter systems to human primary subjects from donors and patients.

## Conclusions

Directed cell migration is a vital biological process in developmental morphogenesis, tissue repair and regeneration, and tumor metastasis. In recent years, cell migration has been drawing increasing attention from physicists and mathematicians, as the directed motion coupled to active deformation is caused by spontaneous symmetry breaking. Quantitative studies of cellular shape changes and active forces yield valuable insights into how genetic mutations or extrinsic cues modulate the logistical delivery of key molecules to functional domains, such as focal adhesion contacts. Currently, most studies have focused on the identification and pathway analysis of proteins involved, whereas the role of lipid membranes has been largely overlooked. This review aimed to highlight the critical roles of lipid membranes in modulating polarization and migration of cells from a biophysical viewpoint.

From a physics viewpoint, extension of the Smoluchowski equation demonstrates that the confinement of key biochemical reactions in quasi-2D membranes increases the efficiency of diffusion-driven reactions. Diffusion in 2D becomes much less dependent on the molecular size when compared with that of the 3D bulk. In a resting cell, proteins regulating cell migration are in the cytoplasm and remain in a non-active, self-inhibited state. Their functions are switched on only after recruitment to the membrane either by electrostatic binding to charged lipid head groups or incorporation of hydrophobic moieties into the membrane core. From a biochemical viewpoint, PI3K is a “master switch” that phosphorylates PIP2 to PIP3 lipids and activates various downstream pathways. Near the leading edge, adhesion domains formed by the phase separation of adhesion molecules in the plasma membrane serve as key reaction centers. Clusters of integrin activate FAK/Src kinase, which recruits PI3K. The “swarm” of PIP3 lipids produced by PI3K logistically recruits key proteins, such as Rho GTPases together with their activators (GEFs) and inhibitors (GAPs), to the plasma membrane and guide their self-organization. Notably, many of the upregulated machineries are not permanent but transient because of negative feedback, which leads to the emergence of stable, oscillatory patterns. For example, oscillatory membrane protrusions near the leading edge are regulated by the antagonistic interplay of RhoA and Rac1, which spatio-temporally coordinates polymerization and crosslinking of actin filaments. Such “membrane-localized reaction hubs” also recruit the plus end of microtubules and hence the centrosome, which defines the global cell polarity that steers cell migration. Thus, lipid membranes are not a physical boundary partitioning cytoplasmic and extracellular spaces or a 2D fluid matrix passively hosting proteins. The logistical transport and transient activation/deactivation of various molecular machineries on the membrane surface is a general principle realizing the robust spatiotemporal control of competing pathways.

## Future perspectives

The regulatory mechanisms of cells are very complex, as partially shown in this review, which often discourages physicists. Nonetheless, if we look into the key molecular processes carefully, we can appreciate that there are physical principles spanning various processes. For example, the use of quasi-2D membranes is a smart strategy designed through evolution that makes the diffusion limited reactions less dependent on the molecular size, while the antagonistic interplay of molecular switches is the basic mechanism that coordinates competing pathways. For example, PI3K/PTEN controls the recruitment/release of Akt to/from cell membranes. The activation of the Akt pathway is essential not only for cell migration but also for cell proliferation, whereas the antagonist PTEN keeps Akt activation by PI3K transient. This negative feedback is essential for the robust control of cell proliferation because permanent overactivation of the Akt pathway causes overgrowth of cells, which leads to tumorigenesis.

In general, physicists tend to describe biological processes by using oversimplified synthetic toy models, which certainly helps to identify the key principles behind complex problems. However, to understand the physical principles that control shape and dynamics of biological cells, it is necessary to investigate real (biological) cells, not only cells from established lines but also primary cells from animal models or human subjects. Modern genetic engineering techniques, such as CRISPR-Cas9 or optogenetic tools, allow us to edit/modify target genes and reveal the key mechanism(s) causing a distinct phenotype. The combination of the control of cell adhesion using extracellular environments, new molecular tools in gene editing, the quantitative analysis of live cell images and the use of quantitative numerical models should help us physically understand how intrinsic and extrinsic cues affect downstream pathways and dynamic cell migration.

## Data Availability

The data and material presented in the manuscript are available from M.T. on request.
